# The whole genome sequence of Coxsackievirus B3 MKP strain leading to myocarditis and its molecular phylogenetic analysis

**DOI:** 10.1186/1743-422X-11-33

**Published:** 2014-02-21

**Authors:** Bin Liu, Zhe Li, Fengyang Xiang, Fan Li, Yang Zheng, Guoqing Wang

**Affiliations:** 1 Cardiovascular disease center, First Hospital of Jilin University, Changchun, Jilin 130021, China; 2 Key Laboratory of Zoonosis, Ministry of Education, Norman Bethune College of Medicine, Jilin University, Changchun, Jilin, China

**Keywords:** Coxsackievirus B3/MKP, Myocarditis, Phylogenetic analysis, Functional genomics

## Abstract

**Background:**

In recent years, the reported infection cases by coxsackievirus (CV) have been on the rise. In order to reveal the relationship between the nucleotide and amino acid sequences and the viral virulence of the CVB3/MKP strain causing myocarditis, we initially confirmed the virulence of the strain in myocardial tissue and then carried out the whole genome sequencing of CVB3/MKP strain and performed a phylogenetic analysis among different CVB3 strains.

**Methods:**

CVB3/MKP infected mouse model was established to check lesions of myocardial tissue in mice using immunohistochemical detection at different periods. RT-PCR analysis was used to amplify seven fragments covering the whole viral sequence and comparable analysis was performed.

**Results:**

The immunohistochemical results showed that particles of CVB3/MKP virus persisted in the cardiac tissue and caused severe pathology. The length of whole genome sequence of CVB3/MKP strain was 7400 bp. CVB3/MKP had 99.7% and 99.6% homology in nucleotide sequence with CVB3/28 and non-virulent CVB3/0, respectively. The former can induce pancreatitis and myocarditis. The nucleotide sequence in the 5′untranslated region of CVB3/MKP strain shared 99.6% and 99.5% homology with CVB3/20 and CVB3/Nancy, respectively.

**Conclusion:**

We confirmed in our animal experiments that CVB3/MKP had cardiotoxicity. CVB3/MKP, CVB3/28, and CVB3/0 may share evolutionary convergence and the 5′untranslated region (5′UTR) may be associated with virulence phenotype. Our findings will provide a basis for identifying the genomic determinant of viral virulence of CVB3/MKP strain and phylogenetic relationship among different CVB3 strains.

## Introduction

In recent years, the reported infection cases by coxsackievirus (CV) have been on the rise and the most commonly known viral infectant of the heart is coxsackievirus B3 (CVB3). It is a member of the genus Enterovirus, which is within the family Picornaviridae. CVB3 RNA, can be detected in the heart muscle of 40–50% of patients with dilated cardiomyopathy (DCM) [1,2]. In addition, epidemiological report of the World Health Organization on the relationship between viral infection and cardiovascular disease indicated that among the 21 viruses which lead to cardiovascular disease, coxsackievirus B3 (CVB3), in particular, is the major pathogen of human viral myocarditis and its infection is widely found in the population, particularly in neonates and young children [3,4]. CVB3 infections cause cardiac arrhythmias and acute heart failure, while in some cases the myocardial inflammation may persist chronically and progress to dilated cardiomyopathy, requiring heart transplantation, or to death [5].

As the whole-genome sequencing techniques have become more advanced in recent years, more and more whole genome sequencing and amino acid sequence for CVB3 strains has been completed [6,7], Some variations in the whole genome of different CVB3 strains can cause certain differences in the structure and function of proteins they encoded, leading to different virulence. A group of reports [8,9] have suggested that CVB3/20, CVB3/28, CVB3/AS and CVB3/Nancy could lead to both pancreatitis and myocarditis, CVB3/CO could only lead to pancreatitis, while CVB3/GA and CVB3/0 were non-pathogenic. Although the different CVB3 strains shared a high degree of sequence identity, even a single nucleotide change in coding or non-coding region of viral genome can cause different tissue tropism and virulence, thereby changing the degree of injury the virus brought on different organs [6,10].

Of note, the 5′untranslated region of CVB3 strains is the main region determining viral virulence, and specific nucleotide variation in the 5′untranslated region may be closely related to different viral virulence, In fact, the mutation of nucleotide U at nucleotide position 234 in the 5′untranslated region of CVB3 strain to C caused its cardiac toxicity significantly reduced. The internal ribosome entry site (IRES), a S-D-like region in configuration and rich in pyrimidine, exists in the 5′untranslated region and binds to ribosome to start viral replication [11], nt88-181 is the region which is a critical determinant of viral myocardial virulence. There was a pre-determined stem-loop structure (SLII) between nt88 and 181 [12], and substitution of the SLII of CVB3/20 with the SLII from CVB3/CO results in a strain which cannot induce myocarditis; but substitution of CVB3/AS or CVB3/20 in the region can restore myocardial virulence of the strain. The group B coxsackievirus type 3 MKP strain leads to myocarditis, but its whole genome sequence has not be acquired. In order to reveal the relationship between the nucleotide and amino acid sequences and the viral virulence of the CVB3/MKP strain causing myocarditis, we first confirmed the virulence of the strain to myocadial tissue and then obtained the whole genome sequence of CVB3/MKP strain using RT-PCR analysis and established a phylogenetic tree among different CVB3 strains. Our findings in this study will lay a foundation for better understanding the genomic features and viral virulence determinant of CVB3/MKP strain and in-depth study of the evolution among different CVB3 strains.

## Results

### CVB3/MKP infection in Swiss mouse myocardium

**Figure 1:**
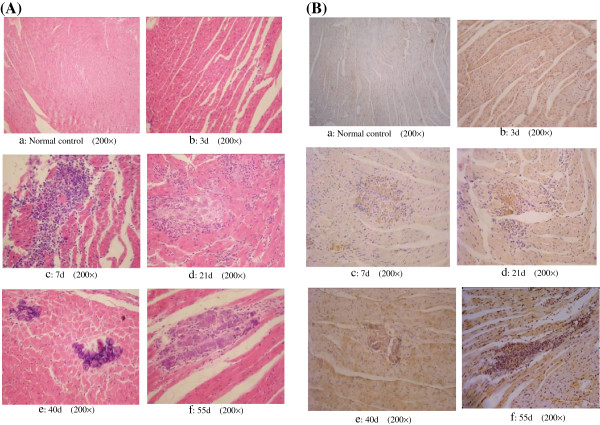
**Cardiac muscle pathological detection of mice infected with CVB3/MKP. (A)** Cardiac muscle of mice infected with CVB3/MKP at different days (H&E staining). Juvenile male Swiss mice were inoculated with virus, and at 3, 7, 21, 40, 55 days post infection, the hearts were removed, sectioned, and stained. The figure shows murine myocardium of negative control (a)*,* and (b) to (f) represent typical histological changes in the heart after infection with CVB3/MKP on different days. **(B)** Immunohistochemistry staining of cardic muscle of mice infected with CVB3/MKP on different days. Anti-CVB3 monoclonal antibody was used. A large number of brown particles existed in the cytoplasm of groups (b) to (f).

The myocardial tissue of mice infected with CVB3/MKP was seriously affected compared with the uninfected mice. In the early viral infection stage, severe congestion appeared in myocardial vascular and interstitial edema was observed with larger fissures. Cloudy swelling appeared in the mouse cardiomyocytes at 7–21 days post infection, with blurred cell stripes, enhanced eosinophilic staining in cytoplasm, condensed fragmented or disappeared nuclei, and spottily distributed infiltrating inflammatory cell, and focal myocardial necrosis was obvious, the infiltrating inflammatory cells in the myocardial tissue was significantly reduced at 40 days post infection, but the myocardial necrosis, calcification of muscle fibers or dissolved absorption still existed (Figure 1A). CVB3/MKP staining and myocardium lesions increased from 3, 7, 21, 40 to 55 days post infection in cardiac tissue shown by immunohistochemistry (Figure 1B), suggesting that the infection may persist in the myocardium for a long-term.

### Characteristics of the whole genome sequence of CVB3/MKP strain and phylogenetic analysis with other CVB3 strains

**Figure 2:**
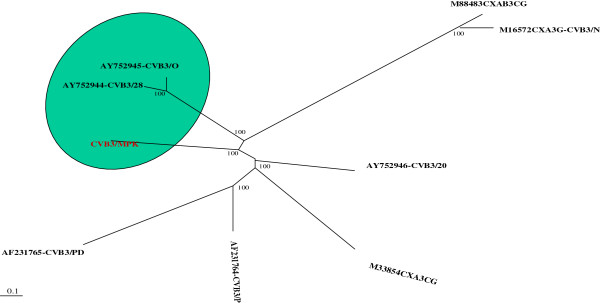
**Phylogenetic tree showing relationship between the complete genomes of CVB3/MKP strain and the other 8 CVB3 strains.** CVB3/MKP is closely related to non-cardiovirulent strain CVB3/0 and cardiovirulent strain CVB3/28.

We obtained the whole genome of sequence CVB3/MKP using RT-PCR method. the complete genome sequence was submitted to GenBank database (Accession number: BankIt1688432,KJ025083). The full-length of the genome was 7400 bp, of which, the length of the open reading frame was 6555 bp that encodes a polyprotein precursor with 2185 amino acids. ClustalW (1.83) software was used to compare CVB3/MKP genome sequence with full-length nucleotide sequences of other eight CVB3 strains in GenBank. The genome sequence identity of the strain with CVB3/28 and CVB3/0 reached 99.7% and 99.6%, respectively. The nucleotide sequence difference between CVB3/MKP and other CVB3 strains was within 0.4% to 0.8% (Table 1). Based on sequence identity analysis, we have established a CVB3 phylogenetic tree in which CVB3/MKP is very close to CVB3/28 and non-pathogenic virus strain CVB3/0. All the three strains were within the same cluster (Figure 2). CVB3/MKP had poor sequence identity with CVB3/Nancy and CVB3 (CXAB3CG), indicating that their phylogenetic relationship was less related.

**Table 1:** Whole genome identity (%) and divergence (%) in nucleotide sequences between CVB3/MKP strain and published CVB3 strains

**Divergence**											
	**1**	**2**	**3**	**4**	**5**	**6**	**7**	**8**	**9**		
**1**		99.6	99.7	99.7	99.7	99.6	99.3	99.6	99.3	**1**	**AF231764-CVB3/P**
**2**	0.4		99.5	99.5	99.5	99.5	99.1	99.5	99.1	**2**	**AF231765-CVB3/PD**
**3**	0.3	0.5		100.0	99.7	99.7	99.3	99.6	99.3	**3**	**AY752944-CVB3/28**
**4**	0.3	0.5	0.0		99.7	99.6	99.3	99.6	99.3	**4**	**AY752945-CVB3/0**
**5**	0.3	0.5	0.3	0.3		99.6	99.2	99.6	99.2	**5**	**AY752946-CVB3/20**
**6**	0.4	0.5	0.3	0.4	0.4		99.2	99.5	99.2	**6**	**CVB3/MKP**
**7**	0.7	0.9	0.7	0.7	0.7	0.8		99.2	100.0	**7**	**M16572CXA3G-CVB3/Nancy**
**8**	0.4	0.5	0.4	0.4	0.4	0.5	0.8		99.2	**8**	**M33854CXA3CG-CVB3**
**9**	0.7	0.9	0.7	0.7	0.7	0.8	0.0	0.8		**9**	**M88483CXAB3CG**
	**1**	**2**	**3**	**4**	**5**	**6**	**7**	**8**	**9**		

Percent identity.

### Sequence identity of 5′UTR nucleotide sequence of CVB3/MKP and phylogenetic analysis with other CVB3 strains

**Figure 3:**
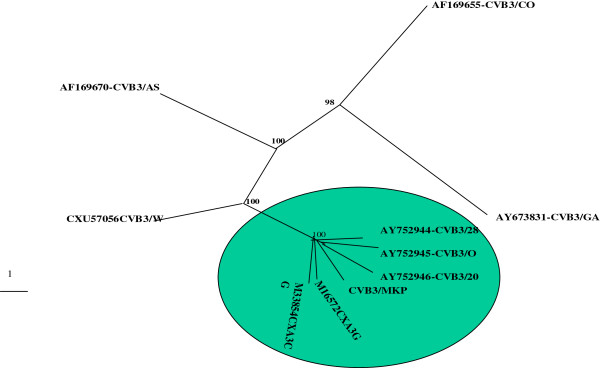
**Phylogenetic relationship among 5′UTR sequences of CVB3 strains.** CVB3/MKP is very closely related to CVB3/20 and CVB3/0, CVB3/28, CVB3/Nancy strains as they form one branch of the tree.

We compared the sequence of the 5′untranslated region of CVB3/MKP with the corresponding sequences of nine CVB3 strains from GenBank, and we found that the sequence identity among CVB3/MKP, CVB3/20, CVB3/28, CVB3/0, CVB3/Nancy, CVB3/AS, CVB3/Woodruff (CVB3/W) and CVB3 (CXAB3CG) was within 91.9?~?99.6% and the sequence identity among CVB3/MKP, CVB3/GA and CVB3/CO were only within 83.3% and 83.6% (Table 2). Based on sequence identity analysis of the above 10 strains in their 5′untranslated region, we established a CVB3 phylogenetic tree in which CVB3/MKP has greater similarity to CVB3/20, CVB3/0, CVB3/28 and CVB3/Nancy, but had less similarity to CVB3/GA and CVB3/CO (Figure 3).

**Table 2:** Identity (%) and divergence (%) in 5′UTR nucleotide sequences between CVB3/MKP strain and published CVB3 strains

**Divergence**											
**1**	**2**	**3**	**4**	**5**	**6**	**7**	**8**	**9**	**10**		
	83.6	83.5	83.7	82.0	80.0	82.2	83.6	83.6	83.5	**1**	**AY673831-CVB3/GA**
20.2		99.9	99.9	92.o	83.2	96.9	99.5	99.6	99.4	**2**	**AY752944-CVB3/28**
20.4	0.1		99.7	91.9	83.0	96.8	99.4	99.5	99.2	**3**	**AY752945-CVB3/0**
20.0	0.1	0.3		92.2	83.3	97.1	99.6	99.7	99.5	**4**	**AY752946-CVB3/20**
23.1	8.5	8.7	8.3		82.0	87.9	91.9	92.0	91.9	**5**	**AF169670-CVB3/AS**
23.5	18.2	18.4	18.0	19.2		81.8	83.3	83.2	83.2	**6**	**AF169665-CVB3/CO**
21.4	3.1	3.3	3.0	9.8	17.1		96.7	96.8	96.8	**7**	**CXU57056B3-CVB3**
20.2	0.5	0.6	0.4	8.7	18.0	3.4		99.5	99.2	**8**	**CVB3/MKP**
20.1	0.1	0.3	0.0	8.4	18.0	2.9	0.4		99.5	**9**	**M16572CXA3G-CVB3/Nancy**
20.2	0.5	0.6	0.4	8.7	18.2	3.1	0.8	0.4		**10**	**M33854CXA3CG-CVB3**
**1**	**2**	**3**	**4**	**5**	**6**	**7**	**8**	**9**	**10**		

### Prediction of RNA secondary structure of SLII region in 5′UTR region of CVB3/MKP

**Figure 4:**
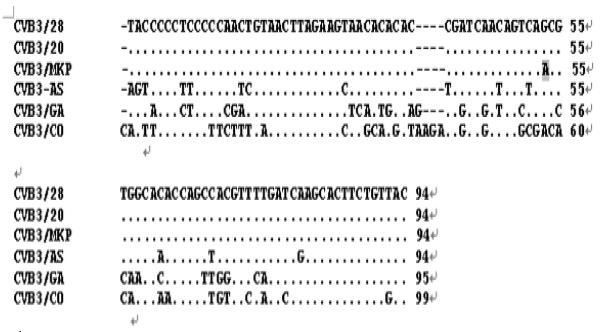
Sequence alignment of stem loop II (SLII) in the 5′non-translation regions of CVB3/20, CVB3/MKP, CVB3/AS, CVB3/GA strains and nt 88–181 of CVB3/28, and nt 88–186 of CVB3/CO.

**Figure 5:**
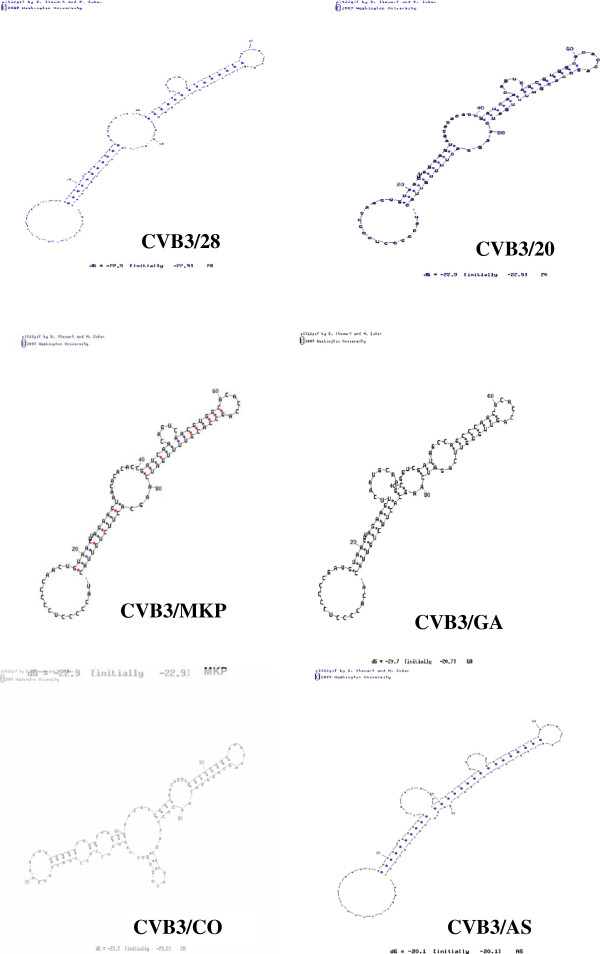
**Predicted RNA secondary structures of stem loop (SL II) region of coxsackievirus B3 (CVB3).** Stem loop (SL II) regions of CVB3/20,CVB3/MKP,CVB3/GA and CVB3/AS strains, nt 88–181 of CVB3/28, and nt 88–186 of CVB3/CO regions were computationally folded.

In order to evaluate the potential role of RNA secondary structure in CVB3 induced myocarditis, we predicted the RNA secondary structure of the SLII region in 5′UTR region of six CVB3 strains CVB3/MKP, CVB3/AS, CVB3/20, CVB3/GA, 88–181 nucleotides of CVB3/28 and 88–186 nucleotides of CVB3/CO using MFOLD program [13]. The results show that the SLII region of CVB3/MKP had only one nucleotide difference from CVB3/20 and CVB3/28 strains, which can induce pancreatitis and myocarditis, respectively, and their RNA secondary structure are basically the same; while the SLII region of CVB3/MKP had 15 nucleotide difference from CVB3/AS strain, they are very similar in their RNA secondary structure. CVB3/CO strain can only induce pancreatitis, and CVB3/GA is a non-pathogenic strain. CVB3/MKP showed great difference from these two strains in terms of SLII nucleotide sequences or RNA secondary structure. The results suggest that the RNA secondary structure of CVB3 may play a role in CVB3 induced myocarditis (Figures 4 and 5).

We then compare the amino acid sequences between CVB3/MKP and CVB3/20, CVB3/28 and CVB3/GA. The results showed that there were only 7 specific variations present in protein coding region, among which 2 sites were located in structural protein and the other 5 in non-structural protein. It was also found that there were 58 variations of amino acids between CVB3/GA and the other 3 strains.

## Discussion

In this study, in order to uncover the relationship between the genome and virulence of CVB3/MKP strain and the phylogenetic relationship between CVB3/MKP strain and other CVB3 strains, we first confirmed that CVB3/MKP strain caused myocarditis and performed a whole genome sequencing of the CVB3/MKP strain and compared its whole genome sequence and the 5′ non-coding region with other strains. We also predicted the RNA secondary structure of the specific function related region and compared the amino acid sequence variation in structural protein region and non-structural protein region.

We confirmed in our animal experiments that CVB3/MKP had cardiactoxicity and the myocardial tissue of mice went through a serious pathological change. This may be ascribed to the bulk replication of CVB3/MKP resulted in necrosis and rupture of myocardium and that CVB antigen triggered auoto-immunresponse via high affinity receptor binding sites of CVB3 antigen [14,15].

The analysis of the whole genome sequence of CVB3/MKP strain revealed that CVB3/MKP shared 99.7% and 99.6% sequence identity with CVB3/28 and CVB3/0 in nucleotide sequence, respectively, and that all the three strains were within the same cluster with a close relationship in evolution. Therefore, the pathogenic strains CVB3/MKP and CVB3/28 may derive from non-pathogenic strain CVB3/0 via mutations under certain conditions. The region bearing small differences among the three strains may have an important role in the formation of viral epitopes, infection and replication of virus. Although the three CVB3 strains shared a high degree of sequence identity, they had different virulence. Therefore, it is possible that small nucleotide variations among the genome of the three CVB3 strains may enable them to have a different tissue tropism or virulence.

Sequence analysis of the 5′untranslated region of CVB3/MKP strain revealed that CVB3/MKP, CVB3/20, CVB3/0, CVB3/28 and CVB3/Nancy were significantly correlated in genetic distance. CVB3/MKP had a longer genetic distance from CVB3/CO and CVB3/GA, which is in accordance with the analysis of the whole genome sequence, further indicating that the pathogenic CVB3/MKP and CVB3/28 strains may derive from the non-pathogenic strain CVB3/0 via mutations under certain conditions. Therefore, the results further proved that the 5′untranslated region of CVB3 may be important for viral tropism and virulence. In addition, sequence identity comparison between CVB3/MKP, CVB3/GA,CVB3/20 and CVB3/28 in amino acid sequences revealed that the amino acid sequences in the coding region of the 4 strains were basically the same, but CVB3/MKP had seven unique mutations, of which, two occurred in structural protein region, five occurred in the non-structural protein region. In the structural protein coding region, the neutralizing antibody sequences in the VP2 and VP3 region of CVB3/MKP strain were substantially similar to other pathogenic CVB3strains, but the 235 amino acid in the VP2 “puff” area of CVB3/MKP strain was Glutamate (E), and the corresponding amino acid in CVB3/GA, CVB3/20 and CVB3/28 was lysine (K); the 512 amino acid in the VP3 “knob” area of CVB3/MKP strain was Valine (V), while the corresponding amino acid of CVB3/GA, CVB3/20 and CVB3/28 was Alanine (A). We also found that non-pathogenic strains CVB3/GA had 58 different amino acids from CVB3/MKP, CVB3/20 and CVB3/28. Although it is not clear whether these variations have any impact on the function of these strains, it is likely that the changes in the nucleic acid or amino acid can influence the ability of CVB3/MKP strain to produce persistent infection. It is necessary to further investigate what role(s) these mutations play in the autoimmune response during the pathogenesis of myocarditis.

In summary, we demonstrated that CVB3/MKP strain had cardiotoxicity and a very close genetic relationship with CVB3/28 strain, which can induce pancreatitis and myocarditis [8,9], and further proved that the 5′untranslated region of CVB3 may be relevant to its cell tropism and virulence phenotypes. Our findings identified the variations in nucleotide and amino acid sequences of the CVB3/MKP strain and a phylogenetic relationship between CVB3/MKP strain and other CVB3 strains, providing a theoretical basis for the pathogenesis and prevention of CVB3-induced myocarditis.

## Materials and methods

### Materials

#### Bacterium strains, cells, viruses, and experimental animals

E. coli JM109 and Hela cells were kept at the Department of Pathogen Biology of Jilin University. CVB3/MKP strain was from Shanghai epidemic prevention station. Healthy, 3-week-old Swiss male mice were obtained from the Experimental Animal Center of Jilin University. The animals were maintained and handled according to standard animal experimental protocol approved by Jilin University.

#### Reagents

Cell culture medium IMDM and fetal calf serum were from Hyclone (USA). Reverse transcriptase Superscript III was from Invitrogen(USA). Taq DNA polymerase, restriction endonuclease Pvu II and pMD18-T vector were from TaKaRa Co (Japan). QIAamp viral RNA Mini Kit was from Qiagen. Mouse anti-CVB3 monoclonal antibody was from Bio Chemicon, Immunohistochemistry kit was from MaiXin New Biotechnology Product Development Company of Fuzhou, China.

### Methods

#### Effect of CVB3/MKP on the myocardial tissue of Swiss mice

80 healthy Swiss mice were randomly divided into CVB3/MKP infection group (group I, n?=?40) in which each mouse was intraperitoneal injected 0.2 ml 400TCID50 CVB3/MKP and normal control group (group II, n?=?40) in which each mouse was intraperitoneal injected 0.2 ml saline, all virus injected mice were separated from normal control mice. At day 3, 7, 21, 40 and 55 post infections, eight mice at each post infection day were sacrificed, and their hearts were removed fixed with 10% formalin and paraffin sectioned, followed by routine hematoxylin-eosin staining (HE staining). For immunohistochemistry, the myocardial sections were treat with 3% H_2_O_2_ at room temperature for 5-10 min, washed with distilled water and PBS for 5 min, and blocked with 5-10% normal goat serum for 10 min. Then, the sections were incubated with mouse anti-CVB3 monoclonal antibody at 4°C overnight, washed with PBS three times, followed by incubation with biotinylated goat anti-mouse secondary antibody at 37°C for 30 min and washed with PBS three times again. Horseradish peroxidase-labeled streptavidin was added to the sections and incubated at 37°C for 30 min; the color was developed with DAB, followed by hematoxylin nucleus staining.

#### Cloning and sequencing of the whole genome of CVB3/MKP strain

After viral replication and amplification, RNA was extracted with Trizol reagent and QIAamp viral RNA Mini Kit, and RT-PCR was performed in a total of 25 μl reaction volume containing cDNA first-strand 1 μl, 10?×?PCR Buffer (Mg2^+^ Plus) 2.5 μl, dNTP Mixture (10 mM) 2 μl, upstream primer CS (10 pmol/μl) 2 μl, downstream primer CS (10 pmol/μl) 2 μl, Taq DNA polymerase (5 u/μl) 0.5 μl, and sterile H_2_O 15 μl. The PCR condition was 94°C for 2 min; 94°C 45 s, 55°C 45 s, 72°C 2 min for 35 cycles; 72°C 10 min. Based on the published sequences from GenBank, seven pairs of primers were designed and synthesized (Table 3). The PCR products were separated and purified by electrophoresis, ligated to pMD18-T vector and positively identified recombinant plasmids were sent to Shanghai Sangon (Shanghai, China) for sequencing.

**Table 3:** Oligonucleotide sequences of primers utilized to amplify the fragments of CVB3/MKP

**Primer name**	**Position**	**Nucleotide sequence (5′-3 ′)**
C1-S	1-20	TTAAAACAGCCTGTGGGTTG
CI-A	1532-1513	GTAGGTTGATCCATTGGTGG
C2-S	1461-1480	TGGTGTATAATGCAGGCATG
C2-A	2477-2458	CTGTTATCGCGTCTTCCACT
C3-S	2411-2431	TTGAAGGACACTCCTTTCAT
C3-A	3342-3322	CTGTAGTTCCCCACATACGC
C4-S	3103-3122	TGCAAGACATGTCAACGCTG
C4-A	3926-3907	CGTGGTTCCTCACCACAATT
C5-S	3844-3863	GGGTCAAGACTCCATCTTAG
C5-A	4543-4524	GTATCCGTCGAAGTGATCTG
C6-S	4518-4538	CAGACCCAGATCACTTCGAC
C6-A	6351-6321	TTGTCCATACATTCCTTTAA
C7-S	6255-6274	GTGCCGGTTACCCATATGTT
C7-A	7400-7387	AATAATCCGCACCGAATGCG

#### Analysis of the whole genome sequence of CVB3/MKP strain

Whole genome sequence was obtained by DNASTAR software. Nucleotide and amino acid sequences analysis and sequence identity comparison with other CVB3 strains from GenBank(Table 4) were completed by NCBI-Megablast and ClustalW (1.83) software [16]. RNA secondary structure was predicted by the MFOLD program (http://mfold.rna.albany.edu/).

**Table 4:** Accession number of reference virus

**Virus**	**Accession number**	**Virus**	**Accession number**
CVB3/GA	AY673831	CVB3/AS	AF169670
CVB3/28	AY752944	CVB3/CO	AF169665
CVB3/20	AY752946	CVB3/W	CXU57056
CVB3/0	AY752945	CVB3/Nancy	M16572
CVB3(CXA3CG)	M33854	CVB3(CXAB3CG)	M88483
CVB3/P	AF231764	CVB3/PD	AF231765

Percent identity.

## Competing interests

The authors declare that they have no competing interests.

## Authors’ contributions

LB carried out the molecular genetic studies, participated in the sequence alignment and drafted the manuscript. LZ carried out the immunoassays. XFY participated in the sequence alignment. LF participated in the design of the study and performed the statistical analysis. WGQ and YZ conceived of the study, and participated in its design and coordination and helped to draft the manuscript. All authors read and approved the final manuscript.
